# Subacute Sclerosing Panencephalitis in Papua New Guinean Children: The Cost of Continuing Inadequate Measles Vaccine Coverage

**DOI:** 10.1371/journal.pntd.0000932

**Published:** 2011-01-04

**Authors:** Laurens Manning, Moses Laman, Henry Edoni, Ivo Mueller, Harin A. Karunajeewa, David Smith, Ilomo Hwaiwhanje, Peter M. Siba, Timothy M. E. Davis

**Affiliations:** 1 School of Medicine and Pharmacology, University of Western Australia, Fremantle Hospital, Fremantle, Western Australia, Australia; 2 Papua New Guinea Institute of Medical Research, Madang, Papua New Guinea; 3 School of Biological, Biomolecular and Chemical Sciences, and School of Pathology and Laboratory Medicine, University of Western Australia, Nedlands, Western Australia, Australia; 4 Pediatrics Division, Modilon General Hospital, Madang, Papua New Guinea; Centers for Disease Control and Prevention, United States of America

## Abstract

**Introduction:**

Subacute sclerosing panencephalitis (SSPE) is a late, rare and usually fatal complication of measles infection. Although a very high incidence of SSPE in Papua New Guinea (PNG) was first recognized 20 years ago, estimated measles vaccine coverage has remained at ≤70% since and a large measles epidemic occurred in 2002. We report a series of 22 SSPE cases presenting between November 2007 and July 2009 in Madang Province, PNG, including localized clusters with the highest ever reported annual incidence.

**Methodology/Principal Findings:**

As part of a prospective observational study of severe childhood illness at Modilon Hospital, the provincial referral center, children presenting with evidence of meningo-encephalitis were assessed in detail including lumbar puncture in most cases. A diagnosis of SSPE was based on clinical features and presence of measles-specific IgG in cerebrospinal fluid and/or plasma. The estimated annual SSPE incidence in Madang province was 54/million population aged <20 years, but four sub-districts had an incidence >100/million/year. The distribution of year of birth of the 22 children with SSPE closely matched the reported annual measles incidence in PNG, including a peak in 2002.

**Conclusions/Significance:**

SSPE follows measles infections in very young PNG children. Because PNG children have known low seroconversion rates to the first measles vaccine given at 6 months of age, efforts such as supplementary measles immunisation programs should continue in order to reduce the pool of non-immune people surrounding the youngest and most vulnerable members of PNG communities.

## Author Summary

Subacute sclerosing panencephalitis (SSPE) is a disabling and usually fatal brain disorder that typically occurs 3–10 years after acute measles infection. Papua New Guinea (PNG) has particularly high rates of SSPE. We report 22 cases of PNG children presenting to the provincial referral hospital in Madang Province who probably contracted acute measles when <12 months of age during a national epidemic in 2002 and who developed SSPE 5–7 years later. Based on these cases, the estimated annual SSPE incidence in Madang province in 2007–2009 was 54/million population aged <20 years. Four sub-districts had an annual incidence >100/million population aged <20 years, the highest rates ever reported. Young PNG children do not respond well to measles vaccine. Because of this, efforts such as supplementary measles immunisation programs should continue in order to reduce the pool of non-immune older people surrounding the youngest and most vulnerable members of PNG communities.

## Introduction

Despite a declining incidence in developed countries, acute measles infection is still responsible for an estimated 164,000 deaths/year and is therefore a major vaccine-preventable cause of death worldwide [1], [2]. Subacute sclerosing panencephalitis (SSPE) is a rare but usually fatal late complication which presents 3–10 years after the acute infection. SSPE is a distinctive clinical entity characterized by behavioural changes and myoclonic jerks, followed by motor dysfunction and profound global cognitive impairment, and then death within a few years of presentation in most cases. The diagnosis is made by the presence of characteristic clinical signs and, if available, electroencephalographic (EEG) findings in conjunction with elevated measles-specific antibodies in serum and cerebrospinal fluid (CSF) [3].

The incidence of SSPE in most countries is <5 per million population <20 years of age, although this figure can be higher in the developing world where vaccination programs are not fully established [4]. The first reports of an unusually high incidence in Papua New Guinea (PNG) were published in the early 1990's [5], with rates between 1988 and 1999 that varied from 13 [5] to 98 [6] per million population <20 years of age. However, these data need to be interpreted against fluctuations in the incidence of acute measles infection over the preceding decade, and should take into account background vaccination coverage and the possibility that localized clusters may contribute disproportionately to overall incidence rates estimated at provincial or country level. In addition, published PNG data to date have come from highland areas which may not be representative of the country as a whole.

We report a series of children presenting to a coastal PNG provincial referral hospital with clinical and laboratory features typical of SSPE. Using available local demographic data, as well as retrospective vaccination and disease surveillance, we have estimated the annual incidence of SSPE in Madang Province and interpreted this figure in relation to prior national measles vaccination coverage and acute measles incidence, as well as the regional distribution of cases.

## Methods

### Ethics statement

Approval for the study was provided by the PNG Institute of Medical Research Institutional Review Board and the Medical Research Advisory Committee of the PNG Health Department. Written informed consent for participation was obtained from parent(s)/guardian(s). The risks and benefits of lumbar puncture (LP) were explained to parent(s)/guardian(s) by the attending ward pediatrician who carried out the procedure with regard for conventional indications (suspicion of meningitis, subarachnoid hemorrhage or central nervous system disease) and contraindications (such as increased intracranial pressure or coagulopathy) [7].

### Study site and patients

Madang Province on the North Coast of PNG has an estimated population of approximately 450,000 people, 54% of whom are <20 years old [8]. Modilon Hospital is the provincial referral hospital and the only health care facility in the province that offers diagnostic and treatment facilities for severely ill patients. A longitudinal detailed observational study of severe illness in all children aged 6 months to 10 years was started at Modilon Hospital at the end of 2006. Prior to this initiative, documentation of cases was insufficient to allow epidemiologic analyses of specific diseases. In November 2007, the first child with symptoms and signs of SSPE was admitted to the present study. There was a subsequent increase in the numbers of similar cases before a decline after 12 months. Data collection was continued until July 2009, at which time relatively few such cases were being admitted.

### National and local measles epidemiology

**Figure 1:**
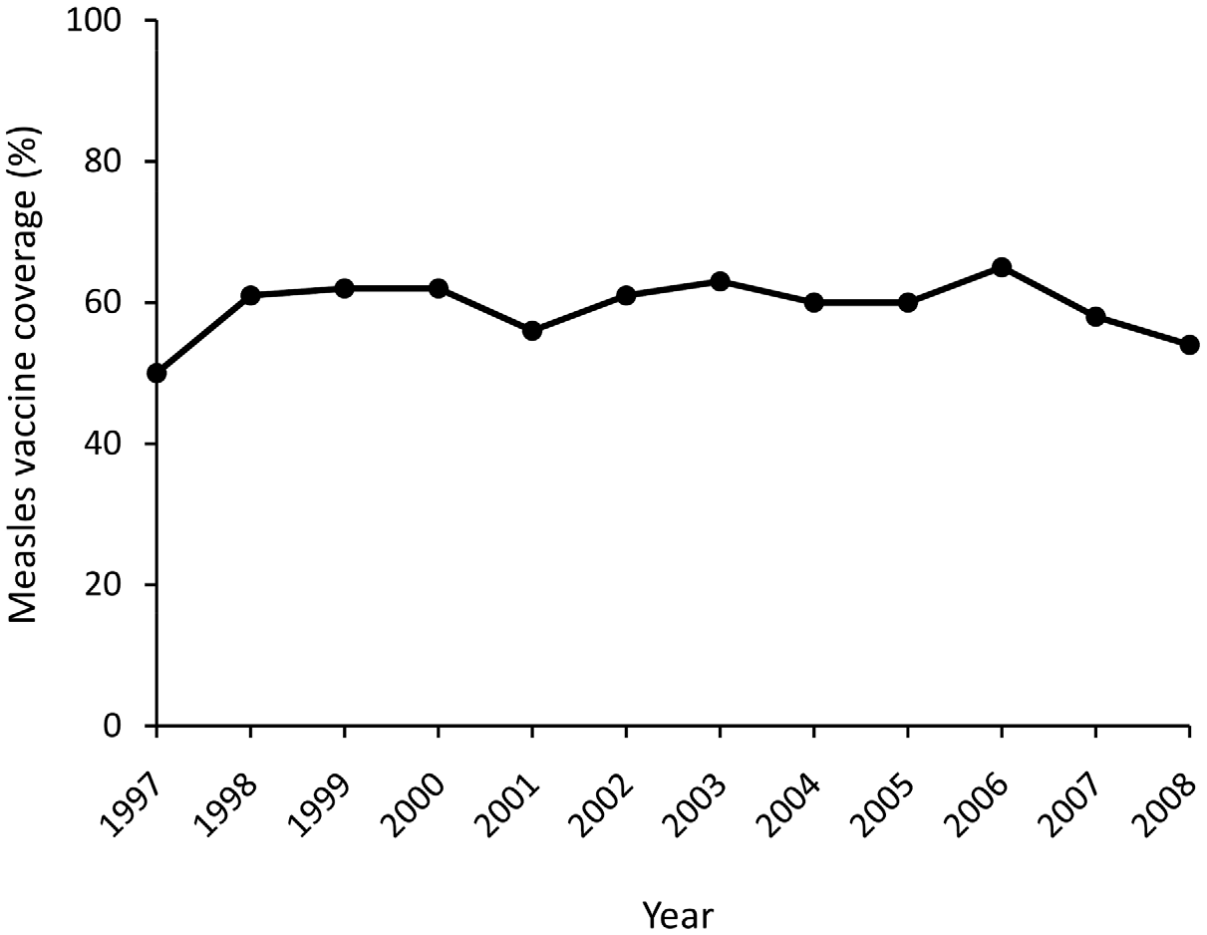
Papua New Guinean national vaccine coverage from 1997 to 2008.

**Figure 2:**
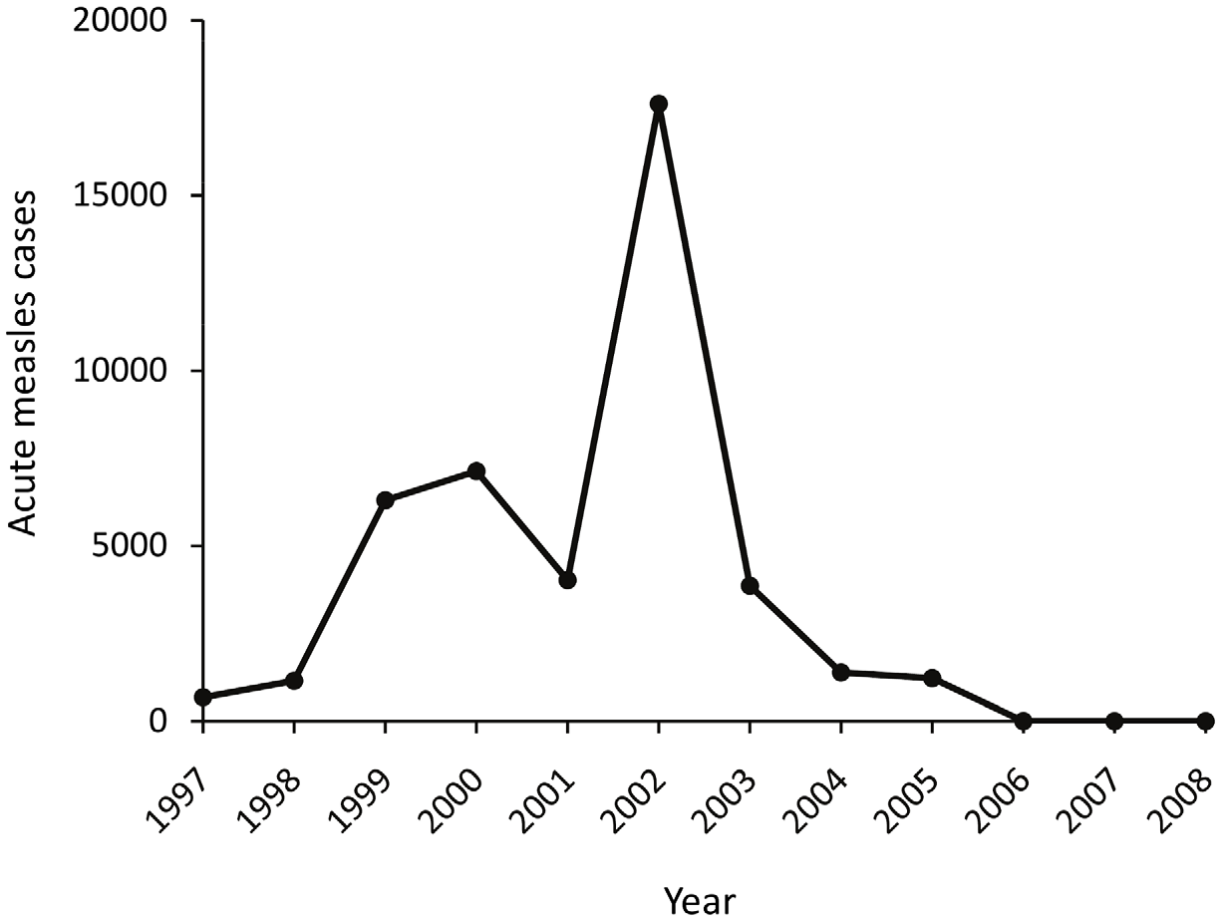
Annual reported numbers of cases of acute measles infection in Papua New Guinea from 1997 to 2008.

Measles immunization was started in PNG in 1982. A modified two-dose schedule at six and nine months of age was used with the aim of providing partial coverage for young infants at high risk of pneumonia and SSPE [9]. However, subsequent available national data indicate that coverage has remained low (see Figure 1). In a recent study of 2007 data, for example, 58% of eligible children received the first dose and 47% the second dose [10]. Cyclical measles epidemics have continued to occur, the last in 2002 (see Figure 2) [11], [12], [13]. Supplementary immunisation activities (SIA) for children aged 6 months to 7 years have been deployed since 2004, with a reported coverage of 79% in 2008 [13].

The measles vaccine coverage recorded in the health diaries of children in Madang Province is similar to that reported elsewhere in PNG, with 41% of children <10 years of age surveyed at two sites within a 20 km radius of Madang town between September 2007 and June 2008 having received at least one dose [10]. Nevertheless, an increasing seroprevalence with age (60% and 79% for children 1–4 years and 5–9 years old, respectively) may indicate that wild measles virus remains prevalent in the community [10] and that there is under-reporting of cases as found in other epidemiologic settings [14], [15].

### Clinical assessment

After recruitment, a standardized case report form was completed detailing demographic information, medical history and history of the current illness. Vaccination history was identified from the health record book held by the parent(s)/guardian(s) of each child where this was available. Since there is no local or central vaccination register, it was assumed that children without such documentation were unvaccinated. Standardized physical assessment included nutritional status assessed by calculating a weight-for-height Z-score [16], with a value <2 considered to indicate malnutrition. We defined severe illness as the presence of one or more of the following features: i) impaired consciousness or coma (Blantyre Coma Score (BCS) <5 [17]), ii) prostration (inability to sit or stand unaided), iii) multiple seizures, iv) hyperlactatemia (blood lactate >5 mmol/L), v) severe anemia (hemoglobin <50 g/L), vi) dark urine, vii) hypoglycemia (blood glucose <2.2 mmol/L), viii) jaundice, or xi) respiratory distress. These criteria are consistent with the World Health Organisation definition for severe malaria [18].

Children with clinical evidence of SSPE, including myoclonic jerks, behavioural changes, and/or speech and motor deficits, underwent detailed neurologic examination by study clinicians (LM, ML). Level of consciousness was graded according to Blantyre Coma Score [17]. Upper motor neuron signs were considered to be present if the child had i) extensor plantar responses, ii) increased muscle tone of either upper or lower limbs, iii) sustained clonus, iv) hyperreflexia, and/or v) pyramidal tract muscle weakness of either upper or lower limbs. In children whose parents/guardians provided informed consent and who had no contraindications, LP was performed. All children were examined daily until discharge at which time a basic assessment of performance status was made. Moderate disability was defined as that requiring considerable assistance with self-care and severe disability as that requiring special assistance with all self-care, categories that are consistent with Karnovsky's performance scores of 50% and <50%, respectively [19].

### Laboratory tests

CSF was examined macroscopically for turbidity, blood staining and clots. We used the Neubauer Improved counting chamber (BoeCo, Germany) to obtain total and differential CSF white cell counts (WCC). Semi-quantitative measures of CSF glucose and protein were performed using dipsticks (Acon Laboratories, San Diego, USA). Specific measles IgG in CSF and serum was measured using a standard indirect immunofluorescence antibody assay (IFA). Serial two-fold dilutions of patient samples were added to separate wells of glass slides to which were fixed measles virus-infected Vero cells. After incubation and washing, anti-human IgG fluorescein isothiocyanate conjugate was then added and, following further incubation and washing, slides were examined under an ultra-violet microscope. Fluorescence was scored as 1+ to 4+, with levels of ≥1+ regarded as positive. This test was performed in an accredited laboratory and had been assessed and approved by the Australian National Association of Testing Authorities in accordance with requirements of the Australian National Pathology Accreditation Advisory Council. Details of other laboratory tests including malaria microscopy, plasma biochemistry and bacterial culture have been published elsewhere [20].

### Case definition of SSPE

Confirmed SSPE was defined as clinical features of SSPE and the presence of measles-specific IgG in CSF, regardless of titer. Probable SSPE was defined as clinical features of SSPE and negative measles-specific IgG in CSF or when no LP was performed.

### Data analysis

The calculation of SSPE incidence was based on PNG Census data for the year 2000 [8] which includes population structure at provincial, district and local-level government (LLG, sub-district) level. The 2008 population was estimated by applying an annual growth rate of 2.6% (Dr Bryant Allen, Australian National University, Canberra, Australia; personal communication). Using this approach, the total population for Madang Province was estimated to be 448,330 with 241,165 (53.8%) <20 years of age. The Global Positioning System co-ordinates of each child's home village were obtained to facilitate LLG incidence estimates [21]. All SSPE incidence rates were expressed per million population <20 years of age which ranged from 5,545 in Iabu Rural LLG to 28,066 in Amenob Rural LLG with an inter-quartile range of 9,880 to 21,267. Reported annual rates of measles vaccination coverage [11], [12] and cases of acute measles infection reported to the PNG Department of Health [12], [13] were obtained from World Health Organization sources. Statistical testing was by means of parametric or non-parametric tests using PASW Statistics (version 17; SPSS Inc. Chicago, Ill) and a level of significance of 0.05.

## Results

### Presenting features and clinical course

Baseline, clinical and laboratory data relating to cases of SSPE identified during the 19-month surveillance period are summarized in Table 1. These 22 children (16 confirmed and 6 probable cases; see below) were a subset of 671 admitted with severe illness during the study period. Although the median duration of illness prior to admission reported by the parent(s)/guardian(s) was 60 (range 1 to 1,000) days, the data provided were not sufficient to allow an accurate estimate of the age of each child at symptom onset.

**Table 1:** Baseline, clinical and laboratory data of the 22 SSPE cases at time of hospital admission.

Demographic details:		
Age (months)	87	(76–95)
Male sex (%)	59	
History of measles (%)	9	
At least one measles vaccination (%)	59	
Clinical signs:		
Malnutrition (%)	16	
Temperature (°C)	36.9	(36.4–37.5)
Blantyre Coma Score ≤4 (%)	55	
Myoclonic jerks (%)	72	
Upper motor neurone signs (%)	72	
Abnormal speech (%)	86	
Laboratory results:		
Lumbar puncture performed (%)	82	
CSF white cells (/µL)	0	[0–20]*
CSF protein (g/L)	0	[0–3.0]*
Serum measles antibody titre	12,228	[16–32,768]
CSF measles antibody titre	128	[0–512]*
Outcome at time of hospital discharge:		
Died (%)	5	
Severe disability (%)	72	

*in the 18 who underwent lumbar puncture.

Data are percentage, mean and (95% confidence intervals), or median and [range].

Two children had documentation or parental knowledge of a past history of acute measles infection, one at six months and the other at two years of age. Neither had a documented history of measles vaccination. There were 14 (64%) children in whom the first dose of measles vaccine had been given and all but one of these (59%) had subsequently received the second dose. In a contemporaneous sample of 44 children hospitalized with other severe non-SSPE illness matched 2∶1 by age and sex with the SSPE cases, the equivalent percentages were 67% and 67% respectively (*P*>0.55 by Chi-squared test). Two children diagnosed with SSPE within a few months of each other were first cousins.

Sixteen children had characteristic myoclonic jerks on admission and four had a clear prior history of myoclonic jerks obtained from the child's parents. One child presented with a short (two-week) history of severe involuntary muscle spasms and died soon after admission, while another presented with complex involuntary dyskinetic movements of upper and lower limbs. The majority of children had additional neurologic findings such as impaired consciousness, difficulty walking and impairment of speech.

LP was performed in 18 of the 22 children. Sixteen of these (89%), including the two with atypical non-myoclonic features, had high titre measles-specific antibodies in both serum and CSF and were therefore confirmed cases of SSPE. Of the probable cases, four did not undergo LP but each had high serum titres of measles-specific antibodies. The remaining two children presented with clinical features consistent with SSPE (myoclonus, motor and speech deficits) with negative CSF measles IgG titers but elevated serum titres at 1∶2048 and 1∶16, respectively. The latter child had no history of measles vaccination.

In all six probable SSPE cases, no other cause of encephalopathy was identified. Normal plasma electrolytes and hepatorenal function excluded metabolic, renal and hepatic encephalopathy. Giemsa-stained thick blood films were negative for malaria parasites and plasma C-reactive protein, blood lactate, white cell count and blood culture results did not suggest an acute infective aetiology. The absence of a CSF pleocytosis in the two children with probable SSPE in whom LP was performed made tuberculous meningitis or cryptococcal meningitis unlikely. CSF from both children was negative by PCR for enteroviruses, Japanese encephalitis virus, Murray Valley encephalitis virus, West Nile virus (including Kunjin) and dengue virus, and serum was negative for the presence of IgM to flaviviruses. Based on clinical presentation and course, serum measles antibody titres and the exclusion of other causes of an encephalopathy, the six children with probable SSPE were included in estimates of SSPE incidence.

Although only one child died in hospital, the remaining children were discharged in line with usual management of SSPE in PNG. These children had moderate or severe disability requiring assistance with most or all activities of daily living. An examination of post-discharge outcome was beyond the scope of the present study.

### Incidence of SSPE in relation to national vaccine coverage and acute measles incidence

**Figure 3:**
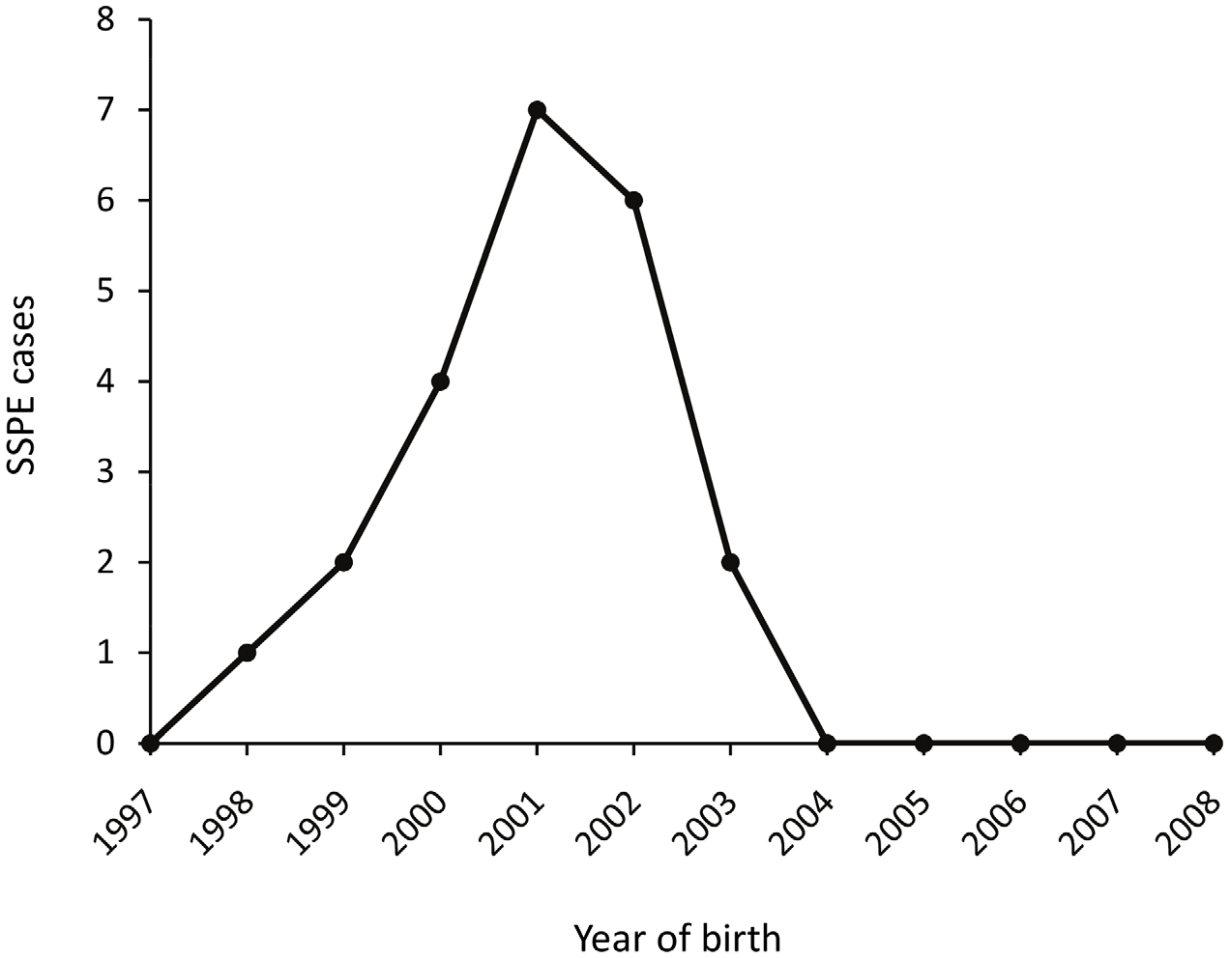
Year of birth of the 22 children with subacute sclerosing panencephalitis (SSPE).

Figures 1 and 2 show PNG national vaccine coverage and acute measles cases since 1997 [11], [12], [13], and the year of birth of the present 22 SSPE cases is shown in Figure 3. Despite relatively stable vaccination coverage between 50% and 65% from 1997 to 2008, there was a substantial increase in the numbers of reported acute measles cases in 2002 with a smaller prior peak in 1999 and 2000. There is a close concordance between the distribution of the years of birth of the SSPE cases and that for acute measles nationally (Spearman r = 0.88, *P* = 0.002).

### Provincial and district incidence of SSPE

**Figure 4:**
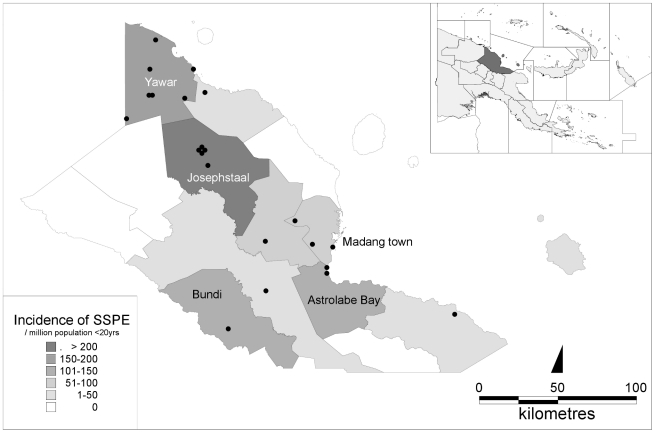
Place of residence of children diagnosed with SSPE in Madang Province during the study, with sub-district-specific annual incidence per million population <20 years of age.

The location of the home village for each child with SSPE and the annual incidence of SSPE in the 13 districts in Madang Province are shown in Figure 4. The majority of the children were from remote rural districts with very limited health care access. The overall estimated annual incidence for Madang province was 29 (95% confidence intervals [18 to 45])/million total population or 54/million population <20 years of age. In Josephstaal, Yawar, Astrolabe Bay and Bundi LLGs, the estimated annual incidence was 296 [96 to 691], 194 [78 to 400], 122 [15 to 442] and 119 [3 to 660]/million, respectively. There were no reported SSPE cases from 4 districts. Three of these have no roads and are only accessible by air, river or foot.

## Discussion

The present study conducted in coastal Madang Province confirms the relatively high incidence of SSPE in PNG shown previously in several highland provincial surveys conducted during the 12 years up to 1999 [5], [6], [22]. However, our data also show that such incidence rates must be interpreted in the light of prior measles epidemiology. There was a clear association between the year of birth of our SSPE cases and national figures for acute measles infection that included a substantial increase in cases in the year 2002. This relationship suggests that, despite the possibility of under-reporting [10], [14], [15], temporal trends in measles cases in PNG are relatively accurate. Without equivalent antecedent data, it is difficult to interpret prior reports [5], [6], [22] in which a high SSPE incidence may have simply reflected peaks in measles cases 3–10 years beforehand. The decline in reported acute measles in PNG since 2002, including very few cases over the last 5 years [13], should herald a substantial reduction in SSPE incidence in PNG over the next few years. Nevertheless, a rising seroprevalence during childhood which exceeds that associated with vaccination coverage and SIA may mean that continued local measles transmission will sustain future low-level presentation of new cases [10]. Although our study captured the delayed peak in SSPE incidence attributable to the 2002 measles epidemic, there have been two further children admitted to Modilon Hospital with a clinical diagnosis of SSPE in the 12 months since recruitment to the present study finished.

The demographic features and clinical course of our patients were similar to those of published series from PNG and other countries. Consistent with previously-reported studies [23], we could not always determine age of onset of symptoms accurately, but the median age at the time of admission in our children (7.3 years) and the male∶female ratio (1.4∶1) were similar to those in SSPE cases from the PNG highlands a decade ago (7.9 years and 1.2∶1, respectively) [4]. Although a male excess is usual, there has been a large age range at presentation, from <5 years in one of the first PNG studies [22] to >10 years in Europid populations [23], [24]. This is likely to reflect population-specific differences in contributing factors such as persistence of maternal antibodies and vaccination policies.

The present study is the first to have had access to LLG population data to facilitate an assessment of SSPE epidemiology at a sub-provincial level in PNG. The incidence of SSPE exceeded 100 per million population <20 years old in four LLGs of Madang Province. Although there were relatively few cases in some of sub-districts, this is the highest rate yet recorded. Only half of PNG children receive both doses of measles vaccine before their first birthday [10], [13], but the prior measles vaccination rate documented for the children from these districts did not differ significantly from that of the non-SSPE severely ill control children nor from national coverage at the time of likely measles infection. This suggests that factors other than vaccine delivery were responsible. There are known continuing difficulties with ensuring a reliable vaccine cold chain in PNG [25], [26], but it is also possible that post-measles vaccination seroconversion rates were unusually low in these areas or that the acute measles incidence was particularly high. Alternatively, the children in these communities have an increased susceptibility to SSPE.

Vaccine seroconversion is highly age-dependent. Only 36% of Melanesian children will develop protective measles immunity after their first vaccination at 6 months [27] while recent data from Madang also indicate low rates of protective immunity to measles in children who had received one or both doses of measles vaccine before one year of age [10]. This reflects, in part, persistence of low-level interfering passive maternal antibodies for up to 12 months [28], especially when maternal immunity has been acquired by natural infection rather than vaccination [29]. The weight of epidemiologic evidence suggests that SSPE is more likely to occur when measles infects a child in the first year of life [23], [30], [31]. Indeed, the clear relationship between year of birth of our children with SSPE and nationally reported measles incidence implies that our cases were very young when they encountered measles virus for the first time. Given that most young children are vulnerable to measles, even if vaccinated, it is likely that differences in the numbers of SSPE cases between districts reflect similar local differences in acute measles incidence between 1998 and 2003.

To ensure adequate herd immunity to measles, countries must achieve 92–95% vaccination coverage that includes two separate doses of the vaccine [32]. Given that the expanded programme for immunisation started in 1982 in PNG and the fact that measles vaccine coverage in PNG has remained ≤70% for at least the last 10 years [11], it is likely that herd immunity was very low in the more remote communities of Madang Province early in the millennium and that acute measles cases were correspondingly high. The fact that four very remote districts were not represented in our series suggests that either they were isolated from the increase in acute measles cases at that time or that children with SSPE were not brought to Modilon Hospital because of the logistic issues involved with patient transfer.

Prior to widespread vaccination, the incidence of SSPE was between 1.2–6.7 per million population <20 years of age in countries where valid data were available [33], [34], [35], [36]. However, incidence rates up to 43 per million population <20 years of age have been estimated in some developing countries [6]. Furthermore, even within closely located communities, the incidence of SSPE is not uniform. For example, Ashkenazi Jews in Israel have a lower rate of SSPE than Separdic Jews (0.5 vs 3.4 cases per million population, respectively) [36]. Our children are from a comparatively homogenous Melanesian group but there were two first cousins of similar age diagnosed within a year of one another. Although from a single set of close-living relatives, SSPE has been described previously in sibling and twin pairs implying at least some familial predisposition, but a clear genetic basis for susceptibility has yet to be defined [37], [38], [39], [40], [41]. Single nucleotide polymorphisms in a number of immunity-related genes have been found to be associated with SSPE in Japanese [42], [43] and Turkish [44] patients, but not in other ethnic groups [45]. The search for genetic associations is difficult in an uncommon disease like SSPE, even in high incidence settings such as PNG, and they would have to account for socio-cultural factors that might promote measles infection at an early age in relatively non-immune, unvaccinated populations [46].

SSPE is diagnosed on clinical grounds alone in resource poor, high incidence settings similar to that of the present study where there are no brain imaging or EEG facilities. Serologic testing for measles is only available as a research or epidemiologic tool. In our case series, six children had probable SSPE without confirmatory CSF serology. Four of these children did not have LP performed but had very high serum titres of measles-specific IgG. The remaining two had negative CSF serology but characteristic clinical features. Because relatively comprehensive clinical and laboratory investigations excluded other likely causes of encephalopathy, the fact that measles CSF and serum titres in SSPE cases can overlap those of controls [3], and given the high pre-test probability of SSPE, we believe that these two latter children represent part of the spectrum of the disease. Our series also included two children with atypical clinical features. One presented with muscle spasms and rapidly progressed to death within two weeks of illness onset. The other had a subacute history and complex dyskinetic movements. Atypical presentations have been described previously and are sometimes accompanied by radiologic evidence of extensive brainstem as well as cortical involvement [47]. It is likely that, had cerebral imaging been available, there would have been similar radiologic findings in these two children.

The PNG Pediatric Guidelines recommend that the first measles vaccine be given at 6 months of age and the second at 9 months of age [7]. The reason for this policy is the increased morbidity and mortality from acute measles in younger infants rather than high rates of SSPE [48]. However, the low seroconversion rates in this age-group argue for a delay in the age of the first measles vaccine to 9 months of age followed by a second dose at 12–15 months. This could be reconsidered if there were to be an outbreak of measles, but this has not happened in PNG for the last 8 years.

The present study extends past published data suggesting that PNG has the highest reported incidence of SSPE globally. However, this high incidence is related to prior measles epidemics. We have also shown that the incidence of SSPE varies between communities. This could reflect localized failure of the vaccine cold chain, community-specific factors that increase measles transmission, variability in public health surveillance and/or differences in genetic susceptibility to SSPE. Young PNG children do not respond well to measles vaccine. Because of this, efforts such as SIA should continue in order to reduce the pool of non-immune older people surrounding the youngest and most vulnerable members of PNG communities. SSPE has a high mortality, but most children with SSPE require prolonged care because of profound disabilities. Such dependence comes at substantial cost for caregivers.

## Supporting Information

Checklist S1 Detailed referencing of STROBE requirements to text of paper
(0.09 MB DOC)
