# Supplementary material for: Subacute Sclerosing Panencephalitis in Papua New Guinean Children: The Cost of Continuing Inadequate Measles Vaccine Coverage
Source: PLoS Negl Trop Dis. 2011 Jan 4;5(1):e932. doi: 10.1371/journal.pntd.0000932 (PMC3014974; doi:10.1371/journal.pntd.0000932)
Supplement: Checklist S1 — Detailed referencing of STROBE requirements to text of paper (0.09 MB DOC) [file pntd.0000932.s001.doc]

STROBE checklist - Detailed referencing of STROBE requirements to text of paper

|  | Item No | Recommendation |
| --- | --- | --- |
| **Title and abstract** | 1 | (*a*) Indicate the study’s design with a commonly used term in the title or the abstract  Observational cohort study design included in Abstract on page 2 |
|  |  | (*b*) Provide in the abstract an informative and balanced summary of what was done and what was found  Provided in Abstract on page 2 |
| Introduction | | |
| Background/rationale | 2 | Explain the scientific background and rationale for the investigation being reported  Included in the Introduction on pages 3 and 4 |
| Objectives | 3 | State specific objectives, including any prespecified hypotheses  Included in the Introduction on pages 3 and 4 |
| Methods | | |
| Study design | 4 | Present key elements of study design early in the paper  Included in the Patients and Methods on pages 5 and 6 |
| Setting | 5 | Describe the setting, locations, and relevant dates, including periods of recruitment, exposure, follow-up, and data collection  Included in the Patients and Methods on pages 5 and 6 |
| Participants | 6 | (*a*) Give the eligibility criteria, and the sources and methods of selection of participants. Describe methods of follow-up  Included in the Patients and Methods on pages 5 and 6 |
|  |  | (*b*) For matched studies, give matching criteria and number of exposed and unexposed |
| Variables | 7 | Clearly define all outcomes, exposures, predictors, potential confounders, and effect modifiers. Give diagnostic criteria, if applicable  Included in the Patients and Methods on page 8 |
| Data sources/ measurement | 8* | For each variable of interest, give sources of data and details of methods of assessment (measurement). Describe comparability of assessment methods if there is more than one group  Included in the Patients and Methods on pages 8 and 9 |
| Bias | 9 | Describe any efforts to address potential sources of bias  Potential bias is addressed in the Results at the top of page 12 and in the Discussion at the top of page 12 |
| Study size | 10 | Explain how the study size was arrived at  Explained under Study site and patients on pages 5 and 6 |
| Quantitative variables | 11 | Explain how quantitative variables were handled in the analyses. If applicable, describe which groupings were chosen and why  Explained in Data analysis section on pages 8 and 9 |
| Statistical methods | 12 | (*a*) Describe all statistical methods, including those used to control for confounding  See in Data analysis section on pages 8 and 9 |
|  |  | (*b*) Describe any methods used to examine subgroups and interactions  See in Data analysis section on pages 8 and 9 |
|  |  | (*c*) Explain how missing data were addressed  See under Presenting features and clinical course on pages 10 and 11 |
|  |  | (*d*) If applicable, explain how loss to follow-up was addressed  Not applicable |
|  |  | (*e*) Describe any sensitivity analyses  Not applicable |
| Results | | |
| Participants | 13* | (a) Report numbers of individuals at each stage of study—eg numbers potentially eligible, examined for eligibility, confirmed eligible, included in the study, completing follow-up, and analysed  Detailed in Results section starting page 9 |
|  |  | (b) Give reasons for non-participation at each stage  Not applicable |
|  |  | (c) Consider use of a flow diagram  Not necessary |
| Descriptive data | 14* | (a) Give characteristics of study participants (eg demographic, clinical, social) and information on exposures and potential confounders  See Results section and Table |
|  |  | (b) Indicate number of participants with missing data for each variable of interest  Detailed in Results section starting page 9 |
|  |  | (c) Summarise follow-up time (eg, average and total amount)  Detailed in Results section starting page 9 |
| Outcome data | 15* | Report numbers of outcome events or summary measures over time  Outcome at discharge is detailed on page10 |
| Main results | 16 | (*a*) Give unadjusted estimates and, if applicable, confounder-adjusted estimates and their precision (eg, 95% confidence interval). Make clear which confounders were adjusted for and why they were included  See Results section, especially paragraph at the top of page 12 |
|  |  | (*b*) Report category boundaries when continuous variables were categorized  Not applicable |
|  |  | (*c*) If relevant, consider translating estimates of relative risk into absolute risk for a meaningful time period  Not applicable |
| Other analyses | 17 | Report other analyses done—eg analyses of subgroups and interactions, and sensitivity analyses  Not applicable |
| Discussion | | |
| Key results | 18 | Summarise key results with reference to study objectives  See first paragraph of the Results section on page 12 |
| Limitations | 19 | Discuss limitations of the study, taking into account sources of potential bias or imprecision. Discuss both direction and magnitude of any potential bias  Limitations are acknowledged in various parts of the Discussion, especially bottom paragraph on page 13 |
| Interpretation | 20 | Give a cautious overall interpretation of results considering objectives, limitations, multiplicity of analyses, results from similar studies, and other relevant evidence  Contained in the Discussion |
| Generalisability | 21 | Discuss the generalisability (external validity) of the study results  Contained in the Discussion |
| Other information | | |
| Funding | 22 | Give the source of funding and the role of the funders for the present study and, if applicable, for the original study on which the present article is based  Provided in the online submission but not in the text, as specified in Instructions to Authors |

*Give information separately for exposed and unexposed groups.

**Note:** An Explanation and Elaboration article discusses each checklist item and gives methodological background and published examples of transparent reporting. The STROBE checklist is best used in conjunction with this article (freely available on the Web sites of PLoS Medicine at http://www.plosmedicine.org/, Annals of Internal Medicine at http://www.annals.org/, and Epidemiology at http://www.epidem.com/). Information on the STROBE Initiative is available at http://www.strobe-statement.org.
